# The relationship between vitamin D levels, D-dimer and platelet parameter levels in patients with gestational hypertension

**DOI:** 10.3389/fimmu.2025.1509719

**Published:** 2025-03-17

**Authors:** Pingping Wang, Jin Yao, Yaqiong Li, Zhanjun Zhang, Ruiling Zhang, Shouting Lu, Meixia Sun, Xiaorong Huang

**Affiliations:** ^1^ Department of Medical Laboratory, Luoyang Maternal and Child Health Hospital, Luoyang, Henan, China; ^2^ Department of Infection and Public Health Management, The Second Affiliated Hospital of Henan University of Science and Technology, Luoyang, Henan, China; ^3^ Medical Imaging Department, Luoyang Maternal and Child Health Hospital, Luoyang, Henan, China; ^4^ School of Humanities and Social Sciences, Luoyang Institute of Technology, Luoyang, Henan, China; ^5^ Luoyang Community Construction and Social Development Research Center, Luoyang Institute of Science and Technology School of Marxism (LIT), Luoyang, Henan, China; ^6^ Research Center of Theoretical Innovation and Think Tank Construction, Luoyang Institute of Science and Technology School of Marxism (LIT), Luoyang, Henan, China; ^7^ Luoyang Research Center for Inheritance and Innovation of Chinese Historical Civilization, Luoyang Institute of Science and Technology School of Marxism (LIT), Luoyang, Henan, China

**Keywords:** gestational hypertension, vitamin D, D dimer, platelet parameters, correlation

## Abstract

**Introduction:**

Hypertension during pregnancy is a common pregnancy complication that has an important impact on maternal and fetal health. In recent years, studies have shown that vitamin D, D dimers and platelet parameters may play a key role in the occurrence and development of gestational hypertension.

**Objective:**

This study aimed to explore the relationship between vitamin D levels, D dimers and platelet parameters in patients with gestational hypertension.

**Material and methods:**

This study retrospectively analyzed the clinical data of 90 patients with gestational hypertension and 90 normal pregnant women who were treated in our hospital from September 2022 to September 2023. We compared the blood routine indicators between the two groups, including platelet count (PLT), mean platelet volume (MPV), platelet distribution width (PDW), etc., as well as D dimer and vitamin D (Vit D) levels.

**Results:**

The results showed that the vitamin D level and PLT in the gestational hypertension group were significantly lower than those in the normal pregnant group, while MPV and PDW were significantly increased. In addition, vitamin D levels were significantly correlated with D dimer, MPV and PDW. Further statistical analysis showed that vitamin D, D dimer and platelet parameters were important predictors of gestational hypertension.

**Conclusion:**

This study found that patients with gestational hypertension have vitamin D deficiency and abnormal platelet function. Vitamin D may affect the development of the disease by regulating platelet activity and coagulation status, which may be closely related to its pathological mechanism. This suggests that improving vitamin D status may have potential value in the management of gestational hypertension.

## 1 Introduction

Hypertensive Disorders of Pregnancy (HDP) is a common disease unique to pregnant women, with an incidence rate between 5% and 10%. HDP is the second leading cause of maternal death after postpartum hemorrhage, accounting for 10% to 16% of total pregnancy-related deaths (1). It is not only an important cause of maternal death, but also a significant factor in neonatal mortality (2, 3). The clinical manifestations of HDP mainly include proteinuria, edema, and hypertension. In severe cases, coma, convulsions, and even death of mother and child may occur (4). At present, the pathogenesis of HDP is not completely clear, but it may be related to immune regulatory function, genetic factors, and abnormal invasion of trophoblast cells (5). Studies have shown that the main pathological changes of HDP include systemic small vessel spasm, reduced organ perfusion, and excessive hypercoagulability (6, 7). The causes of the disease are complex and involve many aspects, such as ischemia and hypoxia caused by shallow implantation of the placenta, immune disorders, very low-density lipoprotein toxicity, genetic imprinting, and large expression of D-dimer. These factors may cause damage to vascular endothelial cells, leading to systemic microvascular spasm and changes in vascular permeability, ultimately causing leakage of body fluids and proteins, redistributing body water and increasing interstitial fluid (8–10). The influencing factors of HDP are diverse and complex, and there are potential risks to both maternal and fetal health. Therefore, it is of great significance to further study the pathogenesis of HDP and its management measures.

Studies in recent years have shown that 25-hydroxyvitamin D3 (25-(OH)D3) is closely related to gestational hypertension (HDP). 25-(OH)D3 has the functions of inhibiting the proliferation of vascular smooth muscle cells, maintaining blood pressure balance and producing anti-inflammatory effects, which makes it play an important role in the prevention of cardiovascular diseases (11). With the development of society and changes in lifestyle, such as increased indoor work and excessive sun protection, the content of vitamin D (VitD) produced by sunlight in the human body has decreased, and the impact of seasons on vitamin D has become smaller and smaller. In China, the proportion of VitD deficiency or insufficiency is about 71%, and among pregnant women, the proportion of severe VitD deficiency is as high as 80% (12), especially pregnant women with a gestational age of more than 30 weeks (13). At present, a serum 25-hydroxyvitamin D concentration below 25nmol/L is usually defined as severe vitamin D deficiency. It is generally believed that the serum 25-hydroxyvitamin D concentration maintained at 50-125nmol/L is a relatively safe range. Within this range, the body’s various physiological functions can be better maintained, which is beneficial to the health of pregnant women and fetuses (14).

VitD deficiency is closely related to the incidence of HDP. VitD has the function of maintaining the stability of vascular endothelial cells, protecting and improving the function of vascular endothelial cells. When VitD deficiency occurs, the content of placental growth factor decreases, the stability and protection of vascular endothelial cells decrease, resulting in the decrease of vascular endothelial anterograde, the increase of hardness, and the increase of both systolic and diastolic blood pressure (15). In addition, VitD can inhibit the renin-angiotensin system, and when VitD is deficient, this inhibition decreases, resulting in the increase of both systolic and diastolic blood pressure in pregnant women (16, 17). Insufficient VitD may also lead to decreased absorption of calcium ions, which can directly affect angiotensin II, and its reduced content can promote angiotensin II to contract vascular smooth muscle function, and eventually lead to preeclampsia like changes in pregnant women (18). *In vivo*, 25-(OH)D3 is the active form of VitD, which can regulate the transcriptional activity of immune cells and up-regulate or inhibit the expression of macrophages, dendritic cells and other cytokines after binding with VitD receptors. When VitD is deficient, the expression of anti-inflammatory factors in pregnant women is reduced, which increases the risk of preeclampsia (19).

In addition, patients with hypertension during pregnancy are often accompanied by obvious hypercoagulable state and platelet activation. Abnormal coagulation function may aggravate the progression of the disease (20). As the disease progresses, vascular endothelial damage intensifies, which not only promotes platelet adhesion and aggregation, but is also accompanied by a decline in microvascular system function, leading to increased platelet destruction. Therefore, the platelet count (PLT) may decrease, while the platelet volume distribution width (PDW) may increase (21). These changes reflect the abnormal characteristics of coagulation function in patients with hypertension during pregnancy. Therefore, understanding and monitoring the levels of VitD, D-dimer and platelet parameters in pregnant women may help prevent and manage HDP early, thereby improving maternal and infant outcomes (22).

Through in-depth understanding and evaluation of the pathogenesis of HDP, it can provide an important theoretical basis for the early diagnosis and effective treatment of the disease. However, despite the progress made in existing research, the complexity and diversity of HDP still need further research and exploration.

## 2 Materials and methods

The subjects of this study were pregnant women with singleton pregnancy who were diagnosed with gestational hypertension in the obstetrics department of a municipal tertiary-level A-class specialist hospital in China from September 2022 to September 2023, with an age range of 25 to 39 years. The diagnostic criteria for hypertensive disorders complicating pregnancy refer to the ninth edition of the textbook of obstetrics and gynecology and the guidelines for the diagnosis and treatment of hypertensive disorders complicating pregnancy (2020). Hypertension complicating pregnancy: hypertension after 20 weeks of pregnancy, systolic blood pressure ≥140 mmHg and (or) diastolic blood pressure ≥90 mmHg, returning to normal within 12 weeks after delivery; urine protein (-); diagnosis can only be made after delivery. A total of 180 pregnant women were included to ensure sufficient statistical power. Exclusion criteria included: genetic metabolic diseases, tumors, vitamin D supplementation in the previous 3 months, and patients taking anticoagulants or platelet inhibitors in the past six months. The control group consisted of pregnant women with singleton pregnancy who had normal prenatal examinations in the obstetrics outpatient clinic of the same hospital, and the exclusion criteria were the same as those of the study group to ensure consistent health conditions. This study was approved by the hospital ethics committee (approval number: KY2022052003.0), and all participants signed informed consent.

The demographic characteristics of each pregnant woman, including BMI and the proportion of primiparas, were recorded through the hospital LIS system. Venous blood was drawn to test CBC, D-dimer and vitamin D levels. CBC was performed using the 6800PLUS fully automatic blood cell analysis system produced by Shenzhen Mindray Company, and blood samples using ethylenediaminetetraacetic acid (EDTA) were collected. The samples were analyzed within 1 hour after collection to prevent platelet swelling and result bias.

Plasma D-dimer was detected using latex turbidimetry, using the Sysmex EX810 fully automatic coagulation analyzer and supporting reagents. Vitamin D (25(OH)D) was determined using chemiluminescence immunoassay, using the Mindray 2000i fully automatic biochemical analyzer and supporting kits produced by Shenzhen Mindray Bio-Medical Electronics Co., Ltd. All measurements were strictly performed in accordance with the manufacturer’s instructions, and corresponding quality control was performed.

### 2.1 Statistical analysis

Statistical analysis was performed using SPSS 25 software. The Kolmogorov-Smirnov normality test was used for the normal distribution of continuous variables. Continuous variables were expressed as mean ± standard deviation (SD) or median (percentile 25, 75) and compared using unpaired Student’s t test or nonparametric Mann-Whitney test. Categorical variables were expressed as counts and percentages and compared using the χ2 test or Fisher’s exact test. Binary logistic regression was used to analyze the influencing factors of gestational hypertension, and ROC curves were used to analyze the diagnostic efficacy of these indicators for gestational hypertension, with P < 0.05 as statistically significant.

## 3 Results

### 3.1 Demographic characteristics and clinical symptoms

A total of 180 pregnant women were included in this study, divided into 90 patients with gestational hypertension (research group) and 90 healthy pregnant women undergoing prenatal care (control group). We compared demographic characteristics such as age, days of gestation, and proportion of primigravida between the two groups. The results showed that there was no significant difference in these basic characteristics between the two groups (see **Table 1**), which laid the foundation for the comparison of subsequent experimental data, allowing the data of different groups to be effectively compared.

**Table 1:** General information comparison of research subjects.

	Age (years)	Pregnancy days	BMI	Primiparous women [cases (%)]
Research group	30.63 ± 4.45	160.24 ± 38.76	58.9 ± 5.2	52 (57.78)
control group	29.77 ± 4.51	163.23 ± 77.75	60.2 ± 4.3	49 (54.44)
t/X2	1.73	-0.574	-0.825	0.207
P	0.086	0.567	0.411	0.648

### 3.2 Laboratory results

**Figure 1:**
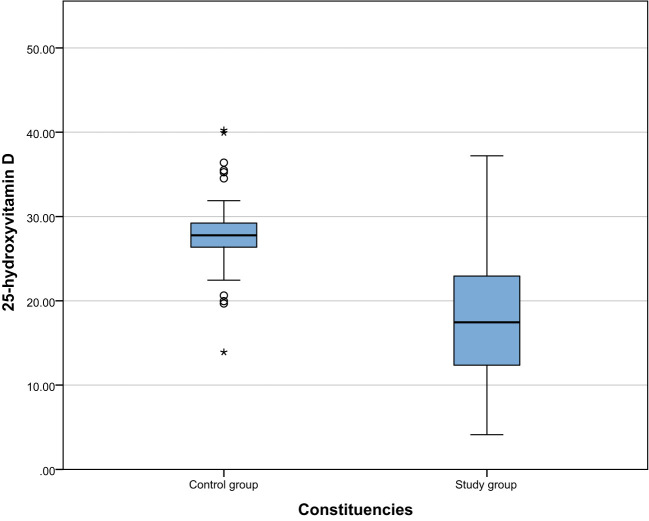
Comparison of 25 hydroxyvitamin D levels between the study group and the control group.

**Figure 2:**
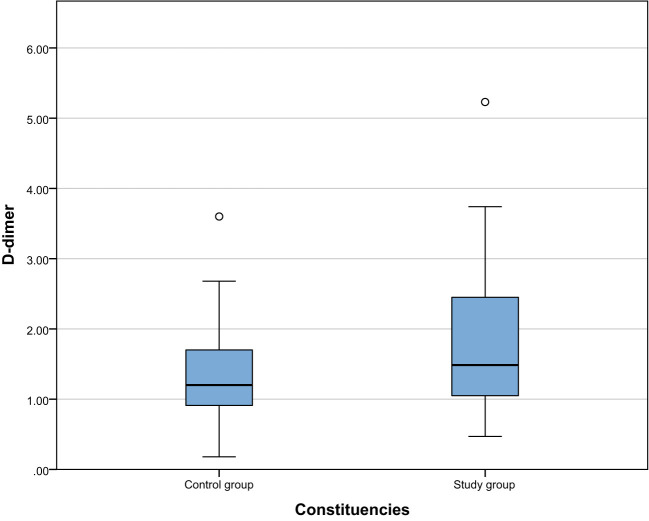
Comparison of PDW between the study group and the control group.

**Figure 3:**
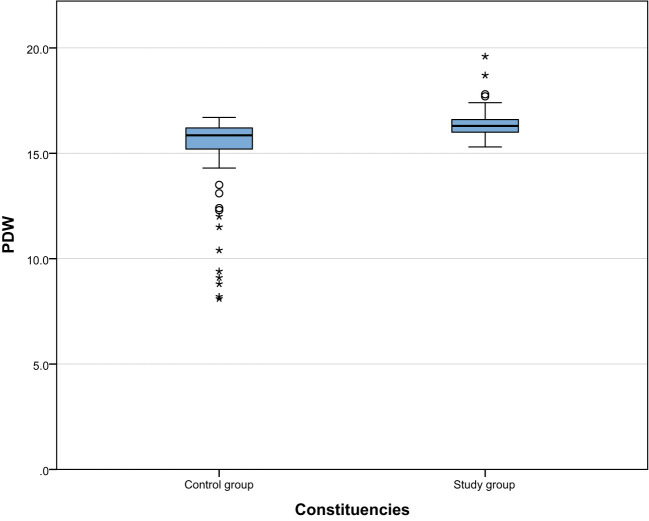
Comparison of PDW between the study group and the control group.

**Figure 4:**
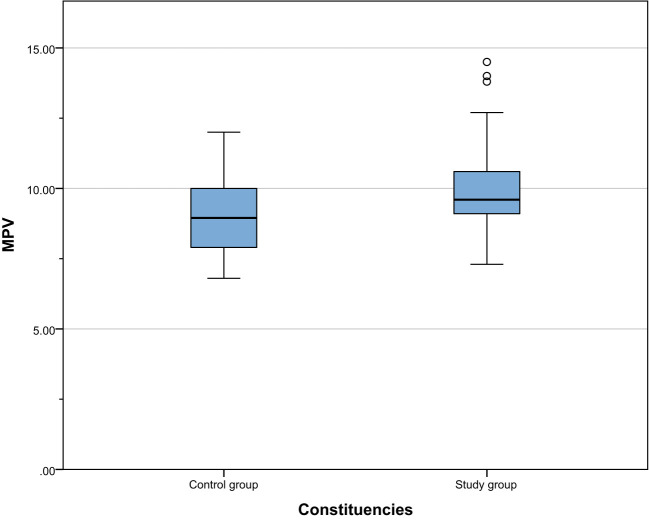
Comparison of MPV between the study group and the control group.

We compared the biochemical indicators of the study group and the control group and found that the 25-hydroxyvitamin D level of the study group was significantly lower than that of the control group [17.46 (12.35, 22.98) vs 27.77 (26.36, 29.24), P < 0.001]. This result shows that vitamin D deficiency is common in patients with gestational hypertension, which may be related to the pathogenesis of gestational hypertension. In addition, the D-dimer ([1.49(1.05,2.46) vs 1.2(0.91,1.7), P=0.002]), PDW ([16.3(16,16.6) vs 15.85(15.18,16.23), P <0.001]) and MPV ([9.6(9.1,10.65) vs 8.95(7.9,10), P<0.001]) were significantly higher than the control group. These results suggest that patients with gestational hypertension not only suffer from vitamin D deficiency, but are also accompanied by platelet function abnormalities and changes in coagulation status, reflecting that the blood system of patients with gestational hypertension may be in a hypercoagulable state. The results are shown in **Table 2**. The comparison between the two groups is shown in **Figures 1**–**4**.

**Table 2:** Comparison of VitD, D-dimer, MPV, and PDW between the study group and the control group.

	Cases no.	25 hydroxyvitamin D ng/mL	D-dimer mg/L	Platelet distribution width %	Mean platelet volume fL
Research group	90	17.46 (12.35,22.98)	1.49 (1.05,2.46)	16.3 (16,16.6)	9.6 (9.1,10.65)
control group	90	27.77 (26.36,29.24)	1.2 (0.91,1.7)	15.85 (15.18,16.23)	8.95 (7.9,10)
Z		-8.498	-3.074	-5.733	-3.771
P		<0.001	0.002	<0.001	<0.001

### 3.3 Correlation analysis

**Figure 5:**
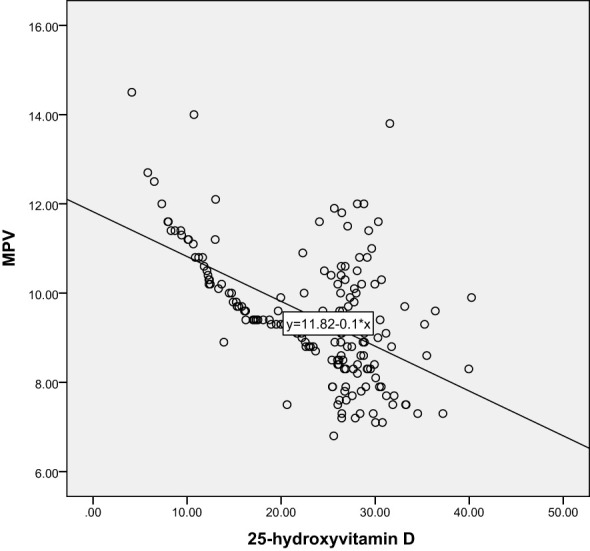
Scatter Plot of the Correlation between 25-Hydroxyvitamin D and MPV.

**Figure 6:**
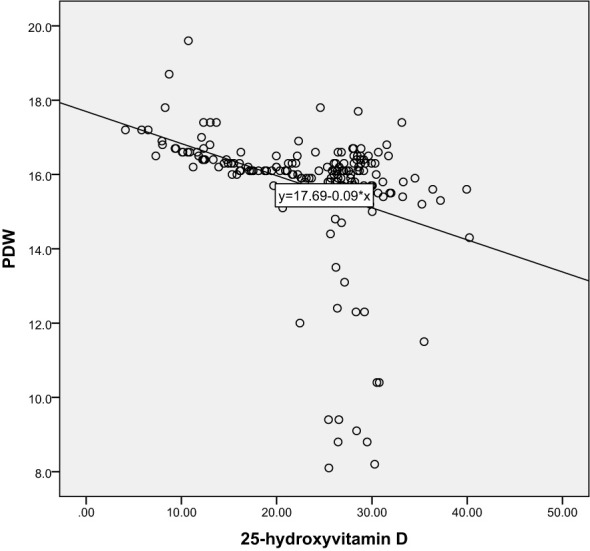
Scatter Plot of the Correlation between 25-Hydroxyvitamin D and PDW.

**Figure 7:**
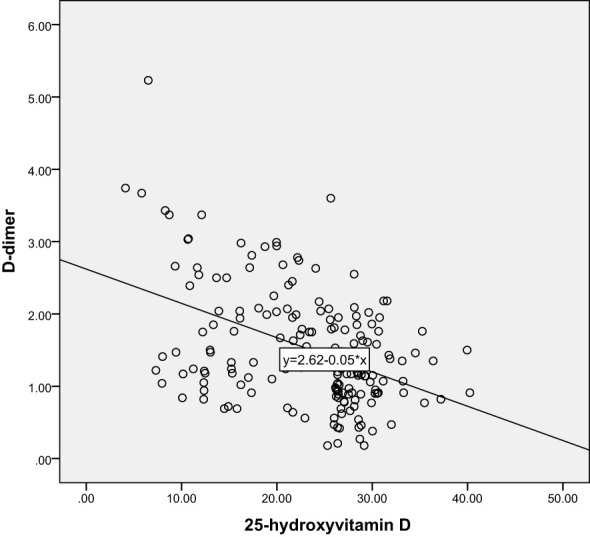
Scatter plot of the correlation between 25-hydroxyvitamin D and D-dimer.

**Figure 8:**
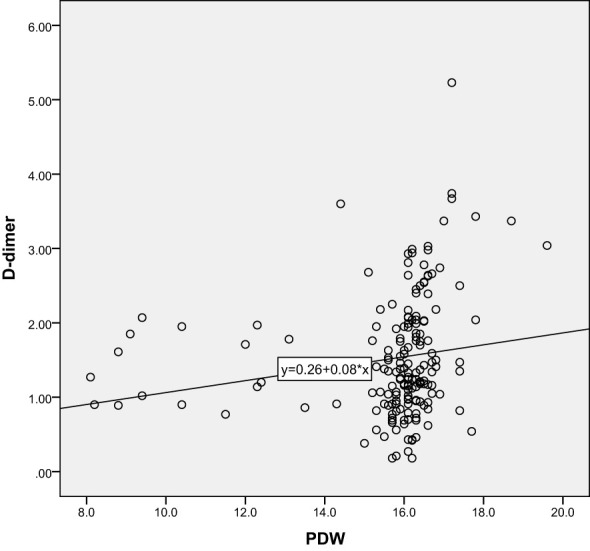
Scatter Plot of the Correlation between D-dimer and PDW.

**Figure 9:**
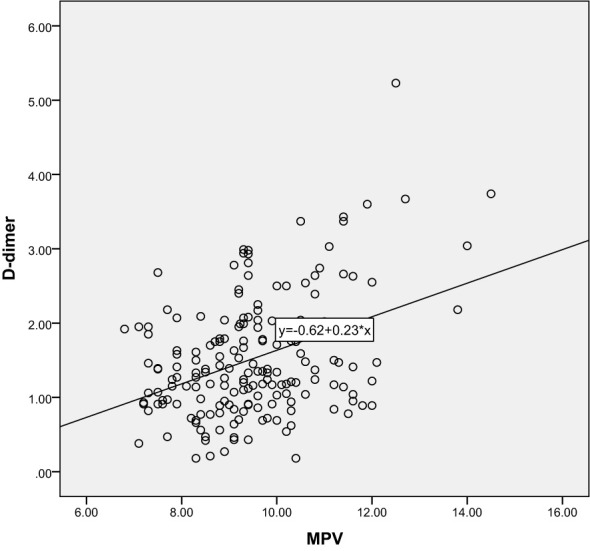
Scatter Plot of the Correlation between D-dimer and MPV.

**Figure 10:**
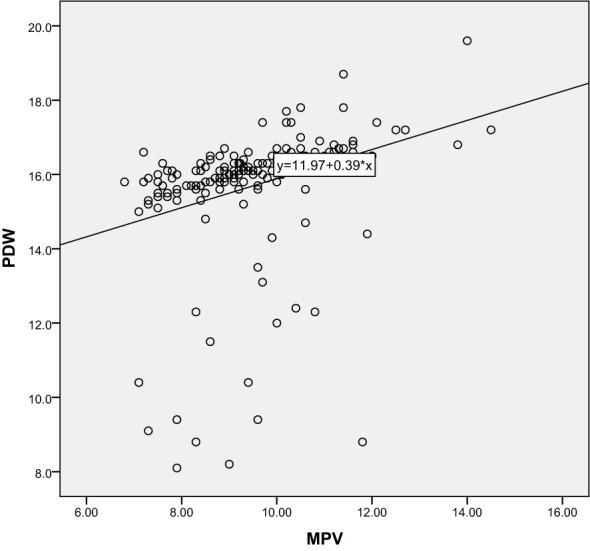
Scatter Plot of the Correlation between MPV and PDW.

Correlation analysis further revealed the relationship between 25-hydroxyvitamin D and other blood indicators. Our results show that there is a significant negative correlation between 25-hydroxyvitamin D and D-dimer (r=-0.365, P<0.001), and a negative correlation with MPV (r=-0.496, P<0.001). There is also a negative correlation with PDW (r=-0.491, P<0.001). These results suggest that reduced vitamin D levels may lead to enhanced platelet activation and coagulation capacity, thereby promoting the development of gestational hypertension. Through these analyses, we can speculate that vitamin D deficiency may be one of the important driving factors for the onset of gestational hypertension. The results are shown in **Table 3** and **Figures 5**–**10**.

**Table 3:** Correlation analysis of 25 hydroxyvitamin D with D-dimer, MPV, and PDW.

		D-dimer	platelet distribution width	mean platelet volume
25-hydroxyvitamin D	correlation coefficient	-0.365**	-0.496**	-0.491**
	P	<0.01	<0.01	<0.01

** indicates that the correlation is significant at the 0.01 level.

### 3.4 Logistic regression analysis

To explore the independent effects of 25-hydroxyvitamin D, D-dimer, PDW and MPV on the occurrence of gestational hypertension, we performed multivariate logistic regression analysis. The results showed that low levels of 25-hydroxyvitamin D (OR=0.43, 95% CI: 0.26-0.72, P=0.002), high D-dimer (OR=2.67, 95% CI: 1.56-4.56, P<0.001), and increased PDW (OR=3.21, 95% CI: 1.86-5.55, P<0.001) and MPV (OR=2.94, 95% CI: 1.68-5.14, P<0.001) were all independent risk factors for gestational hypertension, see **Table 4**. These findings reinforce our previous hypothesis that these biomarkers play an important role in the pathogenesis of gestational hypertension.

**Table 4:** Binary logistic regression analysis of the influencing factors of 25 hydroxyvitamin D, D-dimer, MPV, and PDW on the occurrence of preeclampsia.

	B	Standard error	Wald	Significance	Exp(B)	95% confidence interval of EXP (B)	
						lower limit	upper limit
25 hydroxyvitamin D	-0.296	0.046	41.963	<0.001	0.744	0.68	0.813
D-dimer	0.796	0.221	12.985	<0.001	2.217	1.438	3.419
Platelet distribution width	1.605	0.368	18.99	<0.001	4.977	2.418	10.243
Mean platelet volume	0.468	0.125	13.944	<0.001	1.597	1.249	2.042

### 3.5 ROC curve analysis

**Figure 11:**
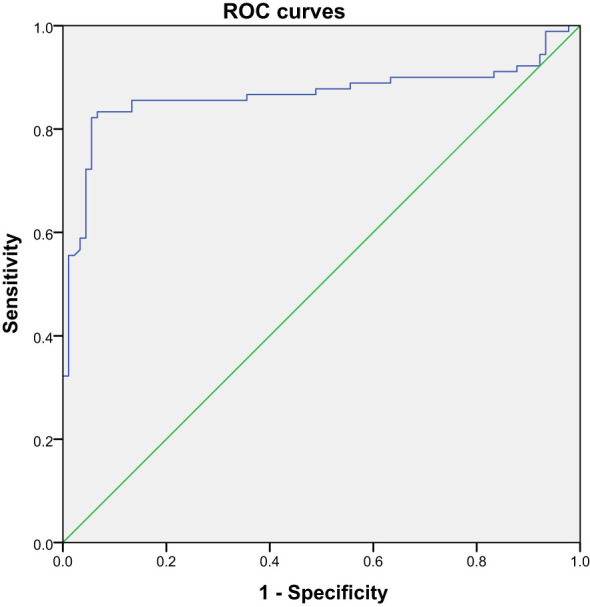
ROC curve analysis of specificity and sensitivity of 25 hydroxyvitamin D.

**Figure 12:**
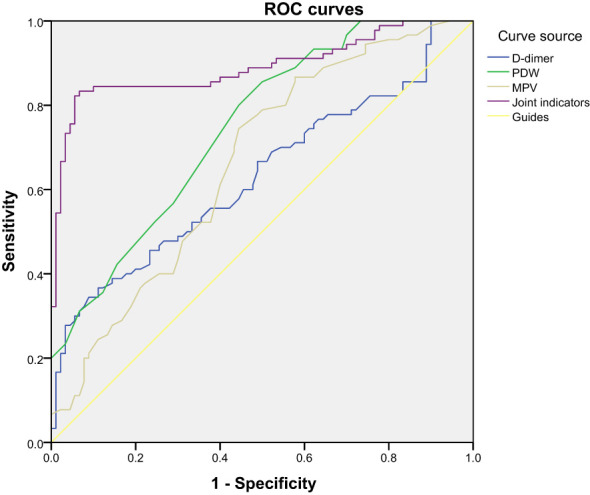
ROC curve analysis of specificity and sensitivity of D-dimer, MPV, and PDW.

Based on our previous results, in order to evaluate the effectiveness of these biomarkers in gestational hypertension screening, we performed ROC curve analysis. The results showed that the AUC of 25-hydroxyvitamin D was 0.82, the AUC of PDW was 0.75, and the AUC of the combined index reached 0.89, indicating that it has good diagnostic ability. In terms of sensitivity and specificity, the performance of the combined index is particularly outstanding, which means that the combination of these indicators in clinical practice can more effectively identify patients with gestational hypertension. The results are shown in **Table 5**, **Figures 11**, **12**.

**Table 5:** ROC curve analysis of the diagnostic value of 25 hydroxyvitamin D, D-dimer, MPV, and PDW for preeclampsia.

Test result variable	region	Cutoff value	Sensitivity	Specificity	Maximum Yoden Index	P	95% asymptotic confidence interval	
							lower limit	upper limit
25 hydroxyvitamin D	0.867	24.95	0.822	0.944	0.766	<0.001	0.805	0.928
D-dimer	0.633	1.98	0.367	0.889	0.256	0.002	0.551	0.714
Platelet distribution width	0.747	15.95	0.8	0.556	0.356	<0.001	0.677	0.817
Mean platelet volume	0.663	9.15	0.744	0.556	0.3	<0.001	0.583	0.742
Merge indicators	0.89	–	0.822	0.944	0.766	<0.001	–	–

## 4 Discussion

Preeclampsia is a serious pregnancy complication with complex etiology. Studies have shown that when the vitamin D level of pregnant women is <50nmol/L, the concentration of placental growth factor decreases, which is not conducive to fetal development; vitamin D can also regulate the activity of immune cells and inhibit the expression of cytokines such as macrophages and monocytes. Pregnant women with gestational hypertension have higher levels of inflammatory factors, and vitamin D can promote the production of anti-inflammatory factors and play a certain anti-inflammatory role (23–25). Vitamin D plays an important role in the etiology and pathophysiology of hypertensive diseases during pregnancy. Our research results support this view, pointing out that there is a clear association between 25-hydroxyvitamin D deficiency and increased coagulation activity and changes in platelet function. Vitamin D deficiency may lead to endothelial cell dysfunction, thereby increasing platelet activation and coagulation tendency, forming a vicious cycle of the development of gestational hypertension. Many studies have shown that vitamin D supplementation during pregnancy can reduce the incidence of hypertensive diseases during pregnancy in people with vitamin D deficiency and is related to the severity of the disease (26, 27), but there is controversy as to whether vitamin D supplementation during pregnancy can improve pregnancy complications and adverse fetal outcomes (28–30).

As a marker of fibrinolysis, elevated levels of D-dimer are usually associated with an increased risk of thrombosis. In our study, the negative correlation between 25-hydroxyvitamin D and D-dimer suggests that low vitamin D levels may lead to overactivation of the coagulation system, thereby increasing the incidence of thrombosis in patients with gestational hypertension. This result is consistent with existing literature (31, 32) and further emphasizes the importance of monitoring D-dimer levels in pregnant women in order to identify potential risks of gestational hypertension early. In addition, HDP patients are often in a chronic diffuse vascular coagulation or prothrombotic state, and dynamic observation of patients’ platelet parameters and coagulation indicators can help reduce the incidence of thrombotic complications (33–35).

Studies have found that vitamin D can inhibit platelet aggregation through endothelial cells. Compared with platelets incubated with untreated endothelial cells, platelets in direct contact with umbilical vein endothelial cells incubated with 1α, 25-dihydroxyvitamin D3 showed lower activation (36). Lower vitamin D levels in the blood are associated with higher platelet reactivity. Lower vit D levels lead to lower VDR expression, thereby reducing PLT activation (37). Our results complement this view. The reduction in 25-hydroxyvitamin D levels is negatively correlated with both MPV and PDW, reflecting the role of vitamin D in regulating platelet function and inhibiting inflammation. Vitamin D deficiency may aggravate the pathophysiological changes of pregnancy-induced hypertension by promoting platelet activation and inflammatory response.

In view of our findings, joint detection of 25-hydroxyvitamin D and other blood indicators can significantly improve the diagnostic rate of gestational hypertension, indicating broad prospects for clinical application. It is recommended that clinicians consider these biomarkers as early screening tools. In this study, the 25-hydroxyvitamin D level in the pregnancy-induced hypertension group was 17.46 (12.35, 22.98) ng/ml. According to the “Crowd VD Deficiency Screening Method (WS/T 677- 2020)” released by China in 2020, serum (or plasma) 25 (OH) D ≥ 20 ng/mL is considered normal VD, 12~<20ng/mL is considered VD insufficiency, and <12ng/mL is considered VD deficiency. It can be seen that the 25-hydroxyvitamin D level in the pregnancy-induced hypertension group is generally insufficient, and some have even reached the level of deficiency, far below the lower limit of the normal level of 20ng/ml, which is a more serious vitamin D deficiency. It is generally believed that the serum 25-hydroxyvitamin D concentration is maintained at 20~50ng/ml as a relatively safe range. Within this range, the body’s various physiological functions can be well maintained, which is beneficial to the health of pregnant women and fetuses. However, the vitamin D levels in the pregnancy-induced hypertension group in this study deviated significantly from this safe range, indicating that vitamin D deficiency is not only common but also severe in patients with pregnancy-induced hypertension. Check pregnant women’s vitamin D levels regularly during pregnancy and supplement when necessary. By improving vitamin D status, it may be possible to reduce the risk of pregnancy-induced hypertension. In addition, our data provide a theoretical basis for vitamin D as a potential target for the prevention and treatment of gestational hypertension, and future clinical trials will help verify the effectiveness of vitamin D supplementation for the prevention and management of gestational hypertension.

The limitations of this study include a relatively small sample size and a cross-sectional design that limits causal inference. To more comprehensively evaluate the relationship between vitamin D and gestational hypertension, large-scale prospective studies should be conducted in the future to validate our findings and explore their potential mechanisms. In addition, the optimal dose of vitamin D supplementation and its efficacy in different populations need to be further explored.

## 5 Conclusion

In summary, the significant correlation between 25-hydroxyvitamin D and gestational hypertension-related blood indicators reveals the important role of vitamin D in hemodynamic regulation during pregnancy. Our study provides a theoretical basis for vitamin D as a new strategy for the prevention and treatment of pregnancy-induced hypertension. Future research should further explore its mechanism and clinical application value.

## Data Availability

The original contributions presented in the study are included in the article/supplementary material, further inquiries can be directed to the corresponding author/s.
